# Effect of race on cardiometabolic responses to once-weekly exenatide: insights from the Exenatide Study of Cardiovascular Event Lowering (EXSCEL)

**DOI:** 10.1186/s12933-022-01555-z

**Published:** 2022-06-27

**Authors:** Timothy M. E. Davis, Anna Giczewska, Yuliya Lokhnygina, Robert J. Mentz, Naveed Sattar, Rury R. Holman, for the EXSCEL Study Group

**Affiliations:** 1 Medical School, University of Western Australia, Fremantle Hospital, P.O. Box 480 Fremantle, WA Australia; 2 Duke Clinical Research Institute, Duke University School of Medicine, Durham, NC USA; 3 Institute of Cardiovascular and Medical Sciences, University of Glasgow, Glasgow, UK; 4 Diabetes Trials Unit, Radcliffe Department of Medicine, University of Oxford, Oxford, UK

**Keywords:** Exenatide once weekly, Cardiovascular risk factors, Racial differences

## Abstract

**Background:**

To determine whether there were racial differences in short-term cardiometabolic responses to once-weekly exenatide (EQW) in the Exenatide Study of Cardiovascular Event Lowering (EXSCEL).

**Methods:**

EXSCEL enrolled 14,752 patients with type 2 diabetes (hemoglobin A_1c_ (HbA_1c_) 6.5–10.0% [48–86 mmol/mol]) with or without cardiovascular disease who were randomized double-blind to EQW or placebo. Background glucose-lowering/other cardiovascular therapies were unaltered for 6 months post-randomization unless clinically essential, facilitating comparison of EQW-associated effects in 14,665 evaluable participants self-identifying as White (n = 11,113), Asian (n = 1444), Black (n = 870), or Other Race (n = 1,238. Placebo-adjusted 6 month absolute changes in cardiometabolic variables were assessed using generalized linear models.

**Results:**

Mean 6-month placebo-adjusted HbA_1c_ reductions were similar in the four groups (range 0.54–0.67% [5.9 to 7.3 mmol/mol], *P* = 0.11 for race×treatment interaction), with no significant difference in Asians (reference) versus other groups after covariate adjustment (all *P ≥* 0.10). Six-month placebo-adjusted mean changes in systolic (−1.8 to 0.0 mmHg) and diastolic (0.2 to 1.2 mmHg) blood pressure, serum LDL (− 0.06 to 0.02 mmol/L) and HDL (0.00 to 0.01 mmol/L) cholesterol, and serum triglycerides (−0.1 to 0.0 mmol/L) were similar in the racial groups (*P* ≥ 0.19 for race×treatment interaction and all *P* ≥ 0.13 for comparisons of Asians with other races). Resting pulse rate increased more in Asians (4 beats/min) than in other groups (≤ 3 beats/min, *P =* 0.016 for race×treatment interaction and all *P* ≤ 0.050 for comparisons of Asians with other races).

**Conclusions:**

Short-term cardiometabolic responses to EQW were similar in the main racial groups in EXSCEL, apart from a greater pulse rate increase in Asians.

*Trial registration*: https://clinicaltrials.gov NCT01144338.

**Supplementary Information:**

The online version contains supplementary material available at 10.1186/s12933-022-01555-z.

## Background

Glucagon-like peptide-1 receptor agonists (GLP-1 RAs) have become established as blood glucose-lowering therapies that should be considered in people with type 2 diabetes if glycemic targets are not met with lifestyle measures and metformin [1], especially in those with, or at high risk of, cardiovascular disease (CVD) [2]. There is some evidence, based on an initial meta-analysis of three Phase IV cardiovascular outcome trials [3], and further supported by a more recent narrative review [4] and an expanded meta-analysis of seven trials [5], that Asians with type 2 diabetes may have fewer major adverse cardiovascular events (MACE; nonfatal myocardial infarction, nonfatal stroke, or cardiovascular death) when treated with GLP-1 RAs than other racial groups. It has been proposed that one of the possible underlying mechanisms for this difference could be a greater improvement in cardiometabolic risk factors resulting from GLP-1 RA therapy in Asian participants [3].

There is, however, inconsistent support for this hypothesis. A systematic review and meta-analysis found that, although there is no evidence of race-specific differences in the pharmacokinetic properties of GLP-1 RAs, the hemoglobin A_1c_ (HbA_1c_) reduction in Asian-dominant studies (those with ≥ 50% Asian participants) was 0.32% (3 mmol/mol) greater than in non-Asian-dominant studies [6]. Subsequent analyses of pooled individual patient data from phase 3 studies have suggested that twice-daily exenatide has greater glycemic efficacy in Asians with type 2 diabetes than other races [7–9], but that there is no such racial difference in the case of once-weekly exenatide (EQW) [8–10]. Race-specific responses of other CVD risk factors, including blood pressure and serum lipids, to exenatide therapy were also assessed in three of these studies, but no formal statistical comparisons were performed [7, 9, 10].

Since available data relating to the glycemic and nonglycemic CVD risk factor response to GLP-1 RA therapy have come largely from people with type 2 diabetes without CVD or at low CVD risk who have participated in Phase III studies, we examined EQW-related changes in cardiometabolic risk factors in participants in the large-scale Phase IV Exenatide Study of Cardiovascular Event Lowering (EXSCEL) [11], with particular reference to those who were Asian.

## Methods

### Study participants and design

EXSCEL was a double-blind, placebo-controlled cardiovascular outcome trial that randomized 14,752 patients with type 2 diabetes, with or without previous cardiovascular disease, to the addition of EQW or placebo to usual care. It showed that EQW was noninferior to placebo when added to usual care for type 2 diabetes with respect to the primary 3-point MACE composite endpoint [11]. Adults aged ≥ 18 years with type 2 diabetes were eligible if their usual care HbA_1c_ was 6.5–10.0% (48 to 86 mmol/mol) inclusive. The EXSCEL protocol specified that ~ 70% of enrolled patients should have previous cardiovascular disease. The trial was run jointly by the Duke Clinical Research Institute and the University of Oxford Diabetes Trials Unit in an academically independent collaboration with the sponsor, Amylin Pharmaceuticals (a wholly owned subsidiary of AstraZeneca) [11].

Participants could be enrolled if treated with a maximum of three oral blood glucose-lowering drugs, or insulin alone or in combination with up to two oral blood glucose-lowering drugs [12]. As part of baseline assessment, participants were asked to self-identify their racial background as Indian (American) or Alaska Native, Asian, Black, Native Hawaiian or Other Pacific Islander, White, Hispanic, or Other [12, 13].

EXSCEL participants were allocated at random in a 1:1 ratio to receive subcutaneous EQW (Bydureon) 2 mg or matching placebo [12]. Other diabetes therapy (addition or substitution of any blood glucose-lowering therapies, including insulin, but excluding GLP-1 RAs) was adjusted by usual care providers according to local management guidelines. Usual care providers were asked to avoid such changes soon after randomization while HbA_1c_ levels were reflecting the initial effect of allocated study medication. Treatment of non-glycemic cardiovascular risk factors was also left to usual care providers with no post-randomization limitations [12].

In this *post hoc* analysis, we aimed to determine whether patients who self-identified as Asian had greater initial reductions in HbA_1c_ and blood pressure, pulse rate, and/or improvements in serum lipid profiles, compared with other racial groups in EXSCEL. CVD risk factors were first reassessed 6 months after baseline when the mean HbA_1c_ difference between the active and placebo groups was maximal because of the initial requirement to keep non-trial blood glucose-lowering therapies stable during this period. This was also the case for other CVD risk factors [11], reflecting the small number of participants in whom adjustments in cardiovascular therapy were made during this period.

### Statistical analysis

Baseline characteristics included demographics, diabetes duration, baseline medications, and medical history. Continuous variables are presented as median and interquartile range (25th, 75th percentiles) and discrete variables as percentages. Fully conditional regression was used to impute missing follow-up and baseline values. Patients with missing racial identification (*N* = 5) and those who died before 6 months (*N* = 82) were not included in analysis. Placebo-adjusted mean absolute changes (mean changes for EQW minus mean change for placebo group) from baseline to 6 months were assessed using generalized linear models with covariates for randomized treatment, race, and race×treatment interaction, adjusted for the baseline value of the outcome variable and clinically plausible confounders including body weight, height, age, statin therapy, beta-blocker therapy, thiazide therapy, use of other open-label glucose-lowering therapies, and smoking status. Models involving blood pressure and pulse rate were further adjusted for a history of heart failure. Mean placebo-adjusted changes and 95% confidence intervals (CI) are shown. All statistical analyses were performed using SAS 9.4 (SAS Institute, Cary, NC). *P*-values < 0.05 were considered statistically significant.

## Results

### Baseline patient characteristics by racial group

Table 1 summarizes the baseline characteristics of the 14,665 evaluable participants (excluding 5 patients with unspecified race and 82 who died before 6 months), categorized by self-identified race. Because of the small numbers in some groups, four race categories were used—White (*N* = 11,113; 75.8%), Asian (*N* = 1444, 9.8%), Black (*N* = 870; 5.9%), with American Indian, Alaska Native, Native Hawaiian or Other Pacific Islander, Hispanic, and remaining racial groups amalgamated into an Other Race category (*N* = 1,38; 8.4%). Asian participants comprised the second largest group after White participants. Compared with the other racial groups, Asians tended to be younger, shorter, and to have a lower BMI. They were also more likely to be treated with a sulfonylurea/other insulin secretagogue or an alpha-glucosidase inhibitor, and they were less likely to have a diagnosis of congestive cardiac failure, to be taking diuretics or ACE inhibitors/angiotensin receptor blockers. They had the lowest median systolic blood pressure along with Other Race, but the highest resting pulse rate. Their serum triglyceride concentrations were comparatively low, but their HbA_1c_ levels and other lipid parameters were similar to those in the other racial groups.

**Table 1:** Baseline characteristics of the trial participants categorized by racial group

	Overall (*N* = 14,665)	White (*N* = 11,113)	Asian (*N* = 1444)	Other Race (*N* = 1238)	Black (*N* = 870)
Age at randomization (years)	62 (56, 68)	63 (57, 69)	60 (54, 66)	61 (55, 68)	60 (53, 66)
Male	62.0%	63.8%	62.3%	54.9%	47.6%
Region					
Europe	46.0%	58.9%	7.8%	1.6%	8.4%
North America	25.1%	25.1%	8.3%	28.6%	49.1%
Latin America	18.4%	13.3%	0.5%	68.5%	42.4%
Asia Pacific	10.4%	2.7%	83.4%	1.3%	0.1%
Diabetes duration (years)	12 (7, 18)	12 (7, 18)	11 (7, 18)	13 (7, 19)	11 (6, 17)
Diabetes therapy (alone/in combination)					
Metformin	76.7%	76.5%	76.5%	80.5%	73.9%
Sulfonylurea/other secretagogue	37.9%	37.0%	44.0%	35.5%	43.1%
Alpha-glucosidase inhibitor	2.0%	1.1%	11.1%	0.7%	0.6%
Thiazolidinedione	3.9%	3.8%	5.1%	3.4%	5.3%
Incretin-based therapies	15.0%	15.9%	17.7%	8.6%	7.7%
Insulin	46.2%	46.1%	47.5%	48.7%	42.1%
Smoking status					
Never/former	88.3%	88.3%	86.6%	92.3%	86.6%
Current	11.7%	11.7%	13.4%	7.7%	13.4%
Prior cardiovascular disease	73.3%	73.0%	77.4%	72.2%	71.7%
Prior coronary artery disease	52.7%	53.5%	58.8%	50.5%	36.6%
Prior cerebrovascular disease	17.0%	17.4%	20.2%	12.0%	13.1%
Prior peripheral arterial disease	18.9%	18.8%	19.9%	19.0%	12.9%
Prior congestive heart failure	16.1%	18.8%	5.4%	8.6%	9.3%
Cardiovascular medications					
Statins	73.5%	74.9%	74.7%	66.2%	63.9%
ACE inhibitors/angiotensin receptor blockers	77.3%	79.3%	67.1%	74.6%	71.7%
Diuretics	43.6%	47.2%	19.9%	34.4%	49.9%
Calcium channel blockers	32.0%	31.7%	39.0%	25.0%	33.3%
Beta blockers	55.7%	58.8%	48.0%	45.2%	43.4%
Aspirin	63.6%	63.9%	61.4%	68.6%	55.9%
Other platelet function antagonists	3.8%	3.4%	7.3%	3.4%	3.2%
Height (cm)	168 (160, 175)	170 (162, 176)	164 (157, 170)	163 (155, 170)	165 (157, 173)
Weight (kg)	90 (77, 103)	94 (82, 108)	71 (62, 81)	80 (69, 93)	88 (75, 103)
Body mass index (kg/m^2^)	31.8 (28.2, 36.2)	32.6 (29.2, 36.9)	26.7 (24.2, 29.7)	30.3 (27.0, 34.2)	31.9 (28.5, 36.7)
Pulse rate (beats/min)	72 (66, 80)	72 (66, 80)	74 (70, 84)	72 (65, 79)	72 (65, 80)
Systolic blood pressure (mmHg)	135 (124, 145)	135 (125, 146)	130 (120, 142)	130 (120, 142)	136 (125, 149)
Diastolic blood pressure (mmHg)	80 (70, 85)	80 (70, 85)	78 (70, 84)	79 (70, 84)	80 (73, 86)
Qualifying HbA_1c_ (mmol/mol)	64 (56, 74)	63 (56, 73)	65 (57, 74)	65 (57, 76)	65 (56, 75)
Qualifying HbA_1c_ (%)	8.0 (7.3, 8.9)	7.9 (7.3, 8.8)	8.1 (7.4, 8.9)	8.1 (7.4, 9.1)	8.1 (7.3, 9.0)
eGFR (mL/min/1.73 m^2^)	75 (61, 90)	74 (60, 89)	76 (60, 94)	75 (61, 92)	83 (67, 99)
Urine albumin:creatinine ratio (mg/mmol)	1.6 (0.5, 5.7)	1.5 (0.5, 5.1)	2.1 (0.5, 10.1)	1.9 (0.4, 7.8)	2.5 (0.5, 10.2)
Serum LDL cholesterol	2.3 (1.7, 3.0)	2.2 (1.7, 3.0)	2.2 (1.7, 2.9)	2.3 (1.7, 2.9)	2.5 (2.0, 3.3)
Serum HDL cholesterol	1.09 (0.91, 1.29)	1.09 (0.90, 1.29)	1.09 (0.90, 1.27)	1.06 (0.91, 1.24)	1.16 (0.98, 1.35)
Serum triglycerides	1.8 (1.3, 2.6)	1.8 (1.3, 2.6)	1.6 (1.2, 2.3)	1.9 (1.3, 2.7)	1.5 (1.1, 2.3)

Data are percentage or median (interquartile range)

### Short-term cardiometabolic response to EQW

There were small reductions in use of individual classes of blood glucose-lowering medications and non-glycemic cardiovascular risk reducing therapies over the first 6 months of follow-up that were similar across the racial groups (see Table 2).

**Table 2:** Medication use of the trial participants at 6 months of follow-up categorized by racial group

	Overall *N* = 14,665	White *N* = 11,113	Asian *N* = 1444	Other races *N* = 1238	Black *N* = 870
Diabetes therapy (alone/in combination):					
Metformin	72.7%	73.0%	70.8%	74.6%	70.3%
Sulfonylurea/other secretagogue	34.9%	34.5%	40.0%	31.4%	37.6%
Alpha-glucosidase inhibitors	1.8%	1.0%	9.7%	0.7%	0.2%
Thiazolidinedione	3.6%	3.4%	4.8%	3.0%	4.4%
Incretin-based therapies	14.0%	14.8%	16.1%	8.9%	8.3%
Insulin	45.0%	45.1%	44.7%	46.5%	41.6%
Cardiovascular medications:					
Statins	68.6%	69.9%	69.9%	60.7%	61.4%
ACE inhibitors or angiotensin receptor blockers	74.3%	76.4%	64.1%	70.0%	71.1%
Diuretics	42.3%	45.6%	20.8%	33.5%	48.4%
Calcium channel blockers	30.7%	30.3%	36.4%	24.7%	34.5%
Beta blockers	53.2%	56.1%	45.6%	43.9%	42.2%
Aspirin	61.2%	61.6%	57.2%	65.0%	58.0%
Other anti-platelet function antagonists	3.3%	2.9%	5.6%	3.2%	4.4%

The 6-month, placebo-adjusted, absolute mean HbA_1c_ change showed no statistically significant treatment effect differences between the four racial groups (*P* = 0.10, Table 3). Mean HbA_1c_ reductions ranged from 5.9 mmol/mol (0.5%) in the White group to 7.3 mmol/mol (0.8%) in the Other Race group. There were similarly no race-specific statistically significant differences in changes in systolic or diastolic blood pressure (*P* ≥ 0.47, Table 3). The 95% CI for the reduction in systolic blood pressure did not include zero for the White, Asian, and Other Race groups, indicating the presence of a treatment effect, but there was no placebo-adjusted reduction in Black participants. There was a consistent mean pulse rate increase of between 2 and 4 beats/min between baseline and 6 months in response to EQW across all racial groups, with the Asians having a significantly greater rise than the other three groups (interaction *P* = 0.016, Table 3). There were no significant EQW effects on serum LDL cholesterol, HDL cholesterol, or serum triglycerides regardless of racial group. Mean reductions in serum LDL cholesterol and serum triglycerides were most pronounced in the White group with the respective 95% CIs not including zero.

**Table 3:** Placebo-adjusted mean (95% confidence interval) changes in cardiometabolic variables from baseline to 6 months by racial group (adjusted models)

Cardiometabolic variable	Racial group	Mean change (95% CI)	Racial difference *P*–value vs. Asians	Overall race × treatment interaction *P*–value
HbA_1c_ (%)	Black	− 0.63 (− 0.77, − 0.46)	0.82	0.10
	Other Race	− 0.66 (− 0.79, − 0.54)	0.80	
	White	− 0.54 (− 0.58, − 0.49)	0.10	
	Asian	− 0.63 (− 0.75, − 0.53)		
HbA_1c_ (mmol/mol)	Black	− 6.8 (− 8.5, − 5.1)	0.82	0.10
	Other Race	− 7.3 (− 8.7, − 5.9)	0.80	
	White	− 5.9 (− 6.4, − 5.4)	0.10	
	Asian	− 7.0 (− 8.3, − 5.8)		
Systolic blood pressure (mmHg)	Black	0.0 (− 2.0, 2.0)	0.19	0.47
	Other Race	− 1.8 (− 3.5, − 0.2)	0.87	
	White	− 1.6 (− 2.1, − 1.0)	0.96	
	Asian	− 1.6 (− 3.2, − 0.1)		
Diastolic blood pressure (mmHg)	Black	1.2 (− 0.1, 2.4)	0.19	0.52
	Other Race	0.7 (− 0.3, 1.7)	0.50	
	White	0.4 (0.1, 0.7)	0.73	
	Asian	0.2 (− 0.7, 1.2)		
Pulse rate (beats/min)	Black	2.1 (0.9, 3.3)	0.006	0.016
	Other Race	2.8 (1.8, 3.8)	0.048	
	White	2.7 (2.4, 3.1)	0.003	
	Asian	4.2 (3.3, 5.1)		
Serum LDL cholesterol (mmol/L)	Black	0.02 (− 0.08, 0.13)	0.27	0.58
	Other Race	− 0.05 (− 0.13, 0.04)	0.89	
	White	− 0.06 (− 0.09, − 0.03)	0.94	
	Asian	− 0.05 (− 0.13, 0.03)		
Serum HDL cholesterol (mmol/L)	Black	0.00 (− 0.03, 0.04)	0.65	0.83
	Other Race	0.01 (− 0.02, 0.03)	0.66	
	White	0.00 (− 0.01, 0.01)	0.36	
	Asian	0.01 (− 0.01, 0.04)		
Serum triglycerides (mmol/L)	Black	0.0 (− 0.2, 0.2)	0.92	0.19
	Other Race	0.0 (− 0.1, 0.2)	0.99	
	White	− 0.1 (− 0.2, –0.1)	0.13	
	Asian	0.0 (− 0.1, 0.2)		

There were similar findings in placebo-adjusted models without further adjustment for confounding variables (see Additional file 1: Table S1), and when absolute changes were analyzed by allocated treatment with and without adjustment for covariates (see Additional file 1: Tables S2 and S3).

## Discussion

These EXSCEL data show that Asians with type 2 diabetes had similar 6-month placebo-subtracted glycemic, blood pressure and serum lipid EQW responses as the other three racial groups studied. Our findings, therefore, confirm that EQW can be used as treatment for type 2 diabetes without regard to race, but call into question the suggestion of a race-specific cardiometabolic contribution to the lower incidence of MACE in Asian participants, as suggested by the meta-analysis of data from GLP-1 RA cardiovascular outcome trials including Liraglutide Effect and Action in Diabetes: Evaluation of Cardiovascular Outcome Results [LEADER], Trial to Evaluate Cardiovascular and Other Long-term Outcomes With Semaglutide in Subjects With Type 2 Diabetes [SUSTAIN-6], and EXSCEL [3, 5]. The rise in pulse rate with EQW therapy was greater among Asians than the other racial groups which might increase their risk of CVD [14], but it might also be a surrogate for greater unmeasured GLP-1 RA responses with CVD benefits in Asians such as anti-inflammatory and antioxidant effects [15].

GLP-1 RAs can be classified as either short-acting with a half-life of a few hours or long-acting with a half-life ≥ 12 h [16]. Long-acting GLP-1 RAs, such as liraglutide, semaglutide, and EQW, are associated with less prandial glucose-lowering efficacy than short-acting compounds such as twice-daily exenatide and once-daily lixisenatide, but they have greater effects on insulin secretion and thus post-absorptive blood glucose concentrations [17]. This distinction was not considered in the meta-analysis, which suggested more potent blood glucose lowering with exenatide in Asians versus non-Asians with type 2 diabetes [6]. However, subsequent analyses of pooled patient-level exenatide data from phase 3 studies have indicated that this benefit is seen with twice-daily but not once-weekly administration [7–10], consistent with the present EXSCEL Phase IV data.

The only other relevant meta-analysis compared three studies in Asians (two with once-daily lixisenatide and one with once-daily liraglutide) to 20 involving White participants treated with GLP-1 RAs ranging from once-daily lixisenatide to weekly semaglutide [18]. Although there was no difference between the two racial groups in HbA_1c_ reduction [18], the analysis did not distinguish between short- and long-acting GLP-1 RAs. In addition, the fact that once daily use of the short-acting lixisenatide means that there is minimal or no drug exposure for most of the period between doses [19] may have attenuated a racial difference, since 82% of the Asians versus 21% of Whites treated with GLP-1 RAs in the analysis were allocated this type of GLP-1 RA therapy [18].

The greater glycemic response of Asians to twice-daily exenatide, compared with EQW, may relate to dietary factors. Compared with Europeans, Asians typically consume more carbohydrates that have a higher glycemic index and which may also lead to a greater subsequent rise in blood glucose [20–22]. This may mean that their response to short-acting GLP-1 RAs with greater postprandial efficacy is also more prominent in type 2 diabetes. An analogous situation is the greater glycemic response of racial groups that have a relatively high carbohydrate intake, including East Asians, to sitagliptin in the Trial Evaluating Cardiovascular Outcomes with Sitagliptin (TECOS) in type 2 diabetes [23], albeit to endogenous incretins rather than GLP-1 RA pharmacotherapy.

There were 2-mmHg mean reductions in systolic blood pressure in racial groups in our study, with the exception of the Black group. This pattern has been reported previously for EQW in smaller groups of participants in Phase III studies, most of which were of similar duration to the present study 6-month follow-up [10]. Mean changes in Phase III studies were approximately 3 mmHg greater in White and Asian racial groups than in those observed in our study [9, 10], and there were also significant reductions in diastolic blood pressure which were not evident in EXSCEL participants. We interpret these differences as reflecting greater use of antihypertensive therapies in our Phase IV study participants with, or at high risk of, CVD, the majority of whom were taking combination blood pressure-lowering therapy at baseline.

As is typical of long-acting GLP-1 RA therapy [24], there were 2–4 beats/min increases in mean pulse rate during the first 6 months of EQW therapy in EXSCEL. The increase was greatest in Asian participants, who also started from a 2 beats/min higher baseline. There is some evidence from general population studies that Asians have a higher resting pulse rate [25], which could reflect a reduced vagal contribution to sympathovagal balance [26] and/or increased sympathetic modulation [27], and perhaps even lower physical activity levels. The former neurohormonal mechanisms have been suggested as causes of the increase in heart rate associated with GLP-1 RAs [28, 29], and it is possible that they mediate the increased pulse rate both before and during EQW therapy in Asians. This aspect of CVD risk has not been examined in the analyses of pooled Phase III data [9, 10], but one study of South Asians with type 2 diabetes treated with liraglutide also suggested an exaggerated pulse rate response to daily liraglutide in this racial group [30].

Although a relatively greater increase in pulse rate resulting from GLP-1 RA treatment would be expected to contribute to an increased CVD risk in Asians versus other racial groups [14], it may also parallel more clinically important enhanced unmeasured responses to GLP-1 RAs that have overriding CVD benefits. There is increasing evidence that GLP-1 may modify CVD risk through direct and indirect actions independent of conventional risk factor changes, including anti-inflammatory and antioxidant activity [15]. It is possible that the greater pulse rate response to EQW in the present study in Asians compared with other racial groups was a surrogate for such larger pleiotropic effects. The limited mediation analyses exploring mechanisms underlying the CVD benefits of GLP-1 RAs performed to date do not appear to have included pulse rate as a candidate variable [31], but this may warrant further consideration.

We found no statistically significant 6-month changes in serum lipid parameters between racial groups in this analysis. Paralleling blood pressure changes, available Phase III data suggest that EQW is associated with significant ~ 0.1 mmol/L reductions in serum LDL cholesterol and triglycerides which are independent of race, including for Asians and Whites [9, 10]. Since almost three-quarters of our participants were treated with a statin at EXCSEL entry, we infer that relatively intensive lipid-modifying therapy in our Phase IV participants masked the modest changes seen in Phase III studies.

There was no evidence of any statistically significant difference in the effect of EQW on the primary MACE endpoint by racial group in EXSCEL [11]. However, in the meta-analyses of LEADER, SUSTAIN-6, and EXSCEL (all involving long-acting analogues), there was a statistically significantly greater CVD benefit of GLP-1 RA therapy in Asians versus Whites (relative risks [95% CI] vs. placebo of 0.35 [0.09, 1.32] and 0.92 [0.73, 1.08], respectively [3], and 0.68 [0.53, 0.84] and 0.87 [0.81, 0.94], respectively [5]). Given the wide confidence intervals and *post hoc* nature of these meta-analyses, this finding should be considered only hypothesis-generating. Aggregate data were utilized, but patient-level data could improve the power and consistency of analyses through better characterization of subgroups [32]. In addition, some potentially confounding variables were not considered. For example, we incorporated smoking in our adjusted models since smoking rates are relatively high in Asian populations [33]. Although smoking is an independent risk factor for CVD [34], and of relevance to treatments such as exenatide as nicotine increases blood glucose levels in a GLP-1-dependent manner [35], it was not considered in the meta-analysis [3].

Our post hoc analyses have limitations. Baseline differences between racial groups in background blood glucose-lowering therapies may have influenced EQW responses during the first 6 months of the study. However, we are not aware of any clinically important relevant interactions. Despite the request for usual care providers to delay therapeutic initial intensification, there may have been small racial differences in changes in lifestyle and pharmacotherapy post-randomization that influenced group-specific cardiometabolic responses leading up to the 6-month follow-up visit. The grouping of participants may have masked important racial and ethnic differences present within the broad regional assignments we used. In the case of Asian participants, who are from an area that covers more than half of the world’s population, we were not able to distinguish between East and South Asians, who are known to have different CVD risks [36]. The strengths of the study are its relatively large number of participants, even relative to previously published meta-analyses, and the incorporation of people with type 2 diabetes at a later stage of their disease compared with participants in Phase III studies from which currently available race-specific comparisons have been made.

## Conclusions

This post hoc analysis of EXSCEL data shows that Asians with type 2 diabetes and CVD or at high cardiometabolic risk have similar glycemic, blood pressure, and serum lipid responses to EQW as other racial groups, including the majority White participants, with the exception of a greater increase in resting pulse rate in Asians. Although it has been suggested in recent meta-analyses that Asians may have greater cardiovascular benefit than White people with type 2 diabetes from long-acting GLP-1 RA therapy [3, 5], the present data do not support race-specific differences in key CVD risk factors as an underlying mechanism. Nevertheless, the cause of race-specific differences in pulse rate and their clinical implications merits further study.

## Supplementary Information

**Additional file 1.** **Table S1.** Race effect on an absolute placebo-adjusted changes in HbA_1c_,blood pressure, heart rate, and serum lipids from baseline to 6 months(unadjusted model). **Table S2.** Race effect on an absolute change in HbA_1c_, blood pressure,heart rate, and serum lipids from baseline to 6 months by treatment (unadjustedmodel). **Table S3.** Race effect on an absolute change in HbA_1c_, blood pressure,heart rate, and serum lipids from baseline to 6 months by treatment (adjustedmodel). 

## Data Availability

The datasets generated during this study and/or as a result of analysis are available from the corresponding author on reasonable request.

## Abbreviations

CI: Confidence intervals
CVD: Cardiovascular disease
EQW: Once-weekly exenatide
EXSCEL: Exenatide Study of Cardiovascular Event Lowering
GLP-1 RA: Glucagon-like peptide-1 receptor agonist
HbA_1c_: Hemoglobin A1c
LEADER: Liraglutide Effect and Action in Diabetes: Evaluation of Cardiovascular Outcome Results
MACE: Major adverse cardiovascular events
SUSTAIN-6: Trial to Evaluate Cardiovascular and Other Long-term Outcomes With Semaglutide in Subjects With Type 2 Diabetes
TECOS: Trial Evaluating Cardiovascular Outcomes with Sitagliptin
