# Supplementary material for: Effect of race on cardiometabolic responses to once-weekly exenatide: insights from the Exenatide Study of Cardiovascular Event Lowering (EXSCEL)
Source: Cardiovasc Diabetol. 2022 Jun 27;21:116. doi: 10.1186/s12933-022-01555-z (PMC9238154; doi:10.1186/s12933-022-01555-z)
Supplement: Supplementary file 1 — Additional file 1. Table S1. Race effect on an absolute placebo-adjusted changes in HbA1c,blood pressure, heart rate, and serum lipids from baseline to 6 months(unadjusted model). Table S2. Race effect on an absolute change in HbA1c, blood pressure,heart rate, and serum lipids from baseline to 6 months by treatment (unadjustedmodel). Table S3. Race effect on an absolute change in HbA1c, blood pressure,heart rate, and serum lipids from baseline to 6 months by treatment (adjustedmodel). [file 12933_2022_1555_MOESM1_ESM.docx]

**Additional file 1: Table S1.** Race effect on an absolute placebo-adjusted changes in HbA_1c_, blood pressure, heart rate, and serum lipids from baseline to 6 months (unadjusted model).

|  |  | **Placebo-adjusted change** | |  |  |
| --- | --- | --- | --- | --- | --- |
| **Cardiometabolic variable** | **Race** | **Mean (SE)** | **95% CI** | **Race Difference *P*-value*** | **Overall interaction *P*-value** |
| HbA_1c_ (mmol/mol) | Black | -6.685 (0.855) | -8.361, -5.009 | 0.6998 | 0.1061 |
|  | Other | -7.291 (0.717) | -8.696, -5.886 | 0.8471 |  |
|  | White | -5.894 (0.239) | -6.362, -5.425 | 0.0866 |  |
|  | Asian | -7.103 (0.664) | -8.404, -5.802 |  |  |
| Systolic blood pressure (mmHg) | Black | -0.050 (1.000) | -2.010, 1.911 | 0.2208 | 0.4981 |
|  | Other | -1.793 (0.839) | -3.436, -0.149 | 0.8662 |  |
|  | White | -1.609 (0.280) | -2.158, -1.060 | 0.9913 |  |
|  | Asian | -1.600 (0.776) | -3.122, -0.078 |  |  |
| Diastolic blood pressure (mmHg) | Black | 1.363 (0.607) | 0.173, 2.553 | 0.1194 | 0.3957 |
|  | Other | 0.703 (0.509) | -0.294, 1.701 | 0.4386 |  |
|  | White | 0.396 (0.170) | 0.063, 0.729 | 0.6470 |  |
|  | Asian | 0.166 (0.471) | -0.757, 1.090 |  |  |
| Pulse rate (beats/min) | Black | 2.192 (0.610) | 0.996, 3.388 | 0.0104 | 0.0244 |
|  | Other | 2.857 (0.512) | 1.855, 3.860 | 0.0595 |  |
|  | White | 2.735 (0.171) | 2.400, 3.069 | 0.0043 |  |
|  | Asian | 4.172 (0.474) | 3.243, 5.100 |  |  |
| Serum LDL cholesterol (mmol/L) | Black | 0.034 (0.054) | -0.072, 0.140 | 0.1704 | 0.3811 |
|  | Other | -0.039 (0.045) | -0.128, 0.049 | 0.7460 |  |
|  | White | -0.062 (0.015) | -0.092, -0.032 | 0.9551 |  |
|  | Asian | -0.059 (0.042) | -0.142, 0.023 |  |  |
| Serum HDL cholesterol (mmol/L) | Black | 0.002 (0.018) | -0.033, 0.037 | 0.5746 | 0.7941 |
|  | Other | 0.007 (0.015) | -0.023, 0.036 | 0.7065 |  |
|  | White | 0.000 (0.005) | -0.009, 0.010 | 0.3345 |  |
|  | Asian | 0.015 (0.014) | -0.013, 0.042 |  |  |
| Serum triglycerides (mmol/L) | Black | 0.021 (0.096) | -0.166, 0.209 | 0.8611 | 0.1691 |
|  | Other | 0.014 (0.080) | -0.143, 0.172 | 0.8992 |  |
|  | White | -0.112 (0.027) | -0.164, -0.059 | 0.1572 |  |
|  | Asian | 0.000 (0.074) | -0.146, 0.146 |  |  |

*Asian race used as a reference group.

**Additional file 1: Table S2.** Race effect on an absolute change in HbA_1c_, blood pressure, heart rate, and serum lipids from baseline to 6 months by treatment (unadjusted model).

|  |  |  | **Absolute change** | |
| --- | --- | --- | --- | --- |
| **Cardiometabolic variable** | **Treatment** | **Race** | **Least Mean Square Estimates** | **95% CI** |
| HbA_1c_ (mmol/mol) | EQW 2 mg | Black | -7.584 | -8.762, -6.405 |
|  |  | Other | -8.837 | -9.821, -7.853 |
|  |  | White | -8.474 | -8.806, -8.141 |
|  |  | Asian | -8.302 | -9.223, -7.381 |
|  | Placebo | Black | -0.898 | -2.090, 0.294 |
|  |  | Other | -1.546 | -2.550, -0.542 |
|  |  | White | -2.580 | -2.911, -2.249 |
|  |  | Asian | -1.199 | -2.118, -0.281 |
| Systolic blood pressure (mmHg) | EQW 2 mg | Black | -1.910 | -3.289, -0.531 |
|  |  | Other | -4.234 | -5.386, -3.082 |
|  |  | White | -2.640 | -3.029, -2.251 |
|  |  | Asian | -3.210 | -4.289, -2.132 |
|  | Placebo | Black | -1.860 | -3.255, -0.465 |
|  |  | Other | -2.441 | -3.616, -1.267 |
|  |  | White | -1.031 | -1.418, -0.644 |
|  |  | Asian | -1.610 | -2.686, -0.535 |
| Diastolic blood pressure (mmHg) | EQW 2 mg | Black | 0.984 | 0.147, 1.821 |
|  |  | Other | -0.939 | -1.638, -0.241 |
|  |  | White | -0.504 | -0.740, -0.268 |
|  |  | Asian | 0.096 | -0.558, 0.750 |
|  | Placebo | Black | -0.379 | -1.226, 0.467 |
|  |  | Other | -1.643 | -2.355, -0.930 |
|  |  | White | -0.900 | -1.135, -0.665 |
|  |  | Asian | -0.070 | -0.723, 0.582 |
| Pulse rate (beats/min) | EQW 2 mg | Black | 3.765 | 2.924, 4.606 |
|  |  | Other | 3.281 | 2.578, 3.983 |
|  |  | White | 3.319 | 3.082, 3.557 |
|  |  | Asian | 6.035 | 5.377, 6.693 |
|  | Placebo | Black | 1.573 | 0.723, 2.424 |
|  |  | Other | 0.423 | -0.293, 1.139 |
|  |  | White | 0.585 | 0.349, 0.821 |
|  |  | Asian | 1.864 | 1.207, 2.520 |
| Serum LDL cholesterol (mmol/L) | EQW 2 mg | Black | -0.034 | -0.109, 0.040 |
|  |  | Other | -0.202 | -0.264, -0.139 |
|  |  | White | -0.145 | -0.166, -0.124 |
|  |  | Asian | -0.133 | -0.191, -0.075 |
|  | Placebo | Black | -0.069 | -0.144, 0.007 |
|  |  | Other | -0.162 | -0.226, -0.099 |
|  |  | White | -0.083 | -0.104, -0.062 |
|  |  | Asian | -0.074 | -0.132, -0.016 |
| Serum HDL cholesterol (mmol/L) | EQW 2 mg | Black | 0.014 | -0.011, 0.038 |
|  |  | Other | -0.019 | -0.040, 0.001 |
|  |  | White | -0.017 | -0.024, -0.010 |
|  |  | Asian | 0.015 | -0.004, 0.035 |
|  | Placebo | Black | 0.012 | -0.013, 0.037 |
|  |  | Other | -0.026 | -0.047, -0.005 |
|  |  | White | -0.017 | -0.024, -0.010 |
|  |  | Asian | 0.001 | -0.018, 0.020 |
| Serum triglycerides (mmol/L) | EQW 2 mg | Black | -0.277 | -0.409, -0.145 |
|  |  | Other | -0.102 | -0.213, 0.008 |
|  |  | White | -0.147 | -0.184, -0.110 |
|  |  | Asian | -0.309 | -0.412, -0.206 |
|  | Placebo | Black | -0.298 | -0.432, -0.165 |
|  |  | Other | -0.116 | -0.229, -0.004 |
|  |  | White | -0.035 | -0.072, 0.002 |
|  |  | Asian | -0.309 | -0.412, -0.206 |

EQW=once-weekly exenatide.

**Additional file 1: Table S3.** Race effect on an absolute change in HbA_1c_, blood pressure, heart rate, and serum lipids from baseline to 6 months by treatment (adjusted model).

|  |  |  | **Absolute change** | |
| --- | --- | --- | --- | --- |
| **Cardiometabolic variable** | **Treatment** | **Race** | **Least Mean Square Estimates** | **95% CI** |
| HbA_1c_ (mmol/mol) | EQW 2 mg | Black | -9.598 | -11.046, -8.149 |
|  |  | Other | -10.716 | -12.032, -9.400 |
|  |  | White | -10.129 | -11.041, -9.217 |
|  |  | Asian | -10.257 | -11.537, -8.978 |
|  | Placebo | Black | -2.792 | -4.248, -1.335 |
|  |  | Other | -3.421 | -4.755, -2.086 |
|  |  | White | -4.238 | -5.147, -3.330 |
|  |  | Asian | -3.208 | -4.483, -1.933 |
| Systolic blood pressure (mmHg) | EQW 2 mg | Black | -1.878 | -3.598, -0.159 |
|  |  | Other | -4.141 | -5.711, -2.572 |
|  |  | White | -2.659 | -3.753, -1.565 |
|  |  | Asian | -2.837 | -4.365, -1.309 |
|  | Placebo | Black | -1.872 | -3.600, -0.144 |
|  |  | Other | -2.306 | -3.894, -0.719 |
|  |  | White | -1.062 | -2.151, 0.026 |
|  |  | Asian | -1.195 | -2.718, 0.328 |
| Diastolic blood pressure (mmHg) | EQW 2 mg | Black | 0.986 | -0.048, 2.020 |
|  |  | Other | -0.539 | -1.482, 0.404 |
|  |  | White | -0.036 | -0.694, 0.622 |
|  |  | Asian | 0.370 | -0.548, 1.288 |
|  | Placebo | Black | -0.258 | -1.297, 0.780 |
|  |  | Other | -1.244 | -2.198, -0.290 |
|  |  | White | -0.446 | -1.100, 0.209 |
|  |  | Asian | 0.128 | -0.787, 1.043 |
| Pulse rate (beats/min) | EQW 2 mg | Black | 2.759 | 1.715, 3.802 |
|  |  | Other | 2.625 | 1.672, 3.577 |
|  |  | White | 2.840 | 2.176, 3.504 |
|  |  | Asian | 5.551 | 4.624, 6.479 |
|  | Placebo | Black | 0.673 | -0.376, 1.722 |
|  |  | Other | -0.214 | -1.177, 0.750 |
|  |  | White | 0.099 | -0.561, 0.760 |
|  |  | Asian | 1.343 | 0.418, 2.268 |
| Serum LDL cholesterol (mmol/L) | EQW 2 mg | Black | -0.085 | -0.175, 0.006 |
|  |  | Other | -0.267 | -0.349, -0.185 |
|  |  | White | -0.120 | -0.177, -0.063 |
|  |  | Asian | -0.200 | -0.279, -0.120 |
|  | Placebo | Black | -0.105 | -0.196, -0.014 |
|  |  | Other | -0.222 | -0.305, -0.139 |
|  |  | White | -0.064 | -0.120, -0.007 |
|  |  | Asian | -0.147 | -0.226, -0.067 |
| Serum HDL cholesterol (mmol/L) | EQW 2 mg | Black | 0.019 | -0.011, 0.048 |
|  |  | Other | -0.041 | -0.068, -0.014 |
|  |  | White | 0.001 | -0.018, 0.020 |
|  |  | Asian | -0.005 | -0.032, 0.021 |
|  | Placebo | Black | 0.014 | -0.016, 0.044 |
|  |  | Other | -0.047 | -0.074, -0.020 |
|  |  | White | 0.000 | -0.019, 0.019 |
|  |  | Asian | -0.020 | -0.046, 0.006 |
| Serum triglycerides (mmol/L) | EQW 2 mg | Black | -0.341 | -0.503, -0.180 |
|  |  | Other | -0.093 | -0.240, 0.054 |
|  |  | White | -0.148 | -0.250, -0.047 |
|  |  | Asian | -0.281 | -0.424, -0.138 |
|  | Placebo | Black | -0.340 | -0.503, -0.178 |
|  |  | Other | -0.106 | -0.255, 0.043 |
|  |  | White | -0.040 | -0.141, 0.062 |
|  |  | Asian | -0.292 | -0.435, -0.150 |
